# Right Heart Failure as an Atypical Presentation of Chronic Type a Aortic Dissection - Multimodality Imaging for Accurate Diagnosis and Treatment. A Case Report and Mini-review of Literature

**DOI:** 10.2478/jccm-2022-0016

**Published:** 2022-08-12

**Authors:** Ioan Tilea, Robert Adrian Dumbrava, Alexandra Mihaela Ratiu, Marius Mihai Harpa, Cosmin Marian Banceu, Dorina Nastasia Petra, Horatiu Suciu

**Affiliations:** 1 George Emil Palade University of Medicine, Pharmacy, Science, and Technology of Targu Mures, Targu Mures Romania; 2 Department of Internal Medicine II- Cardiology II, Emergency Clinical County Hospital, Targu Mures, Romania; 3 Department of Radiology and Medical Imaging, Emergency Clinical County Hospital, Targu Mures, Romania; 4 Cardiac Surgery Clinic, The Emergency Institute for Cardiovascular Diseases and Transplantation, Targu Mures, Romania

**Keywords:** right heart failure, pericardial hematoma, chronic type A aortic dissection, multimodality imaging, cardiac surgery

## Abstract

**Background:**

An intrapericardial organized haematoma secondary to chronic type A aortic dissection is an extremely rare cause of right heart failure. Imaging studies are essential in recognising and diagnosis of this distinctive medical condition and guiding the anticipated treatment.

**Case presentation:**

A 70-year-old male patient was admitted for progressive symptoms of right heart failure. His cardiovascular history exposed an aortic valve replacement 22 years before with a Medtronic Hall 23 tilting valve with no regular follow-up. Classical signs of congestion were recognized at physical examination. Transthoracic two-dimensional echocardiography and thoraco-abdominal computed tomography angiography, as essential parts of multimodality imaging algorithm, established the underlying cause of right heart failure. Under total cardiopulmonary bypass and cardiac arrest, surgical removal of the haematoma and proximal repair of the ascending aorta with a patient-matched vascular graft were successfully performed. The patient was discharged in good condition with appropriate pharmacological treatment, guideline-directed; no imagistic signs of acute post-surgery complications were ascertained.

**Conclusion:**

This paper highlights the importance of recognizing and providing a timely clinical and imagistic diagnosis of this very rare, potentially avoidable cause of right heart failure in patients with previous cardiac surgery.

## Introduction

The normal structure of the aorta can be altered by different congenital or acquired diseases [1]. Aneurysmal arterial disease is distinct from atherosclerosis and other occlusive arterial diseases in terms of its clinical factors, histologic findings, and mechanistic qualities [2]. During the progression of undiagnosed and/or untreated aortic aneurysms, severe, potential deadly complications such as dissection and rupture can occur. Aortic dissection represents a life-threatening condition caused by a tear in the intimal layer of the aorta or bleeding inside the aortic wall, with heterogenous and overlapping clinical manifestations [3,4]. Aortic dissection can be divided into an acute phase (<14 days), followed by a subacute phase up to 3 months from symptom onset, as well as chronic (>3 months) [1,4,5]. The Stanford classification divides aortic dissection into type A (TAAD), which involves the ascending aorta, and type B, when the dissection begins beyond the take-off point of the left subclavian artery [6]. The incidence of aortic dissection was reported by a European multicentric study to be 6 per 100,000 people per year, with 71% being TAAD [7]. Ascending aorta dissections are less common, with an incidence of approximately 3.5 per 100,000 people per year [8]. The incidence rate of chronic aortic dissection is assumed to be between 4 and 6 per 100,000 per year [9].

The main risk factors for aortic dissection development include male sex; advanced age; conditions associated with increased aortic wall stress such as hypertension, trauma, and cocaine use; smoking; a high apolipoprotein B/A1 ratio; conditions associated with aortic media abnormalities (genetic disorders such as Marfan, Ehlers–Danlos, Loeys–Dietz, and Turner syndromes); and previous aortic diseases [2,10, 11, 12, 13]. Chronic TAAD may occur as a result of delayed diagnosis and/or surgical interventions in patients with acute TAAD, dilatation of the aorta distal to the aortic graft after the surgical treatment of acute TAAD, or previous heart surgery [14,15]. Interestingly, in patients with prior cardiac surgery, some studies suggest that the symptoms of chronic aortic dissection are partially alleviated, with patients being more clinically and haemodynamic stable - possibly due to mediastinal adhesions, which can protect against cardiac tamponade [16,17].

Angiographic computed tomographic scanning, which is widely used, provides typical imagistic signs of chronic TAAD as a non-wavy, fixed and stiff intimal flap, which confirms the full extent of the disease and suggests the need for appropriate planning by the cardiac surgeon [5,18].

A careful evaluation of cases with a high probability of TAAD permits a personalized, patient-centred medical and surgical approach for each case, as a delay in diagnosis and treatment is associated with increased mortality [5].

In this paper, we present the case of a 70-year-old male patient with a previous surgical aortic valve replacement (AVR) who developed progressive chronic heart failure (HF) secondary to a TAAD complicated with a massive intrapericardial hematoma compressing the right atrium, right pulmonary artery, and superior and inferior vena cava. The pericardial hematoma was peeled off from the pericardium and surface of the heart with concomitant successful proximal repair of the ascending aorta from the sino-tubular junction to below the origin of the brahiocephalic trunk, with a 30 mm-diameter vascular graft.

## Case Presentation

A 70-year-old Caucasian man, a former heavy smoker, was admitted to a general surgery department to cure a right inguinal hernia, despite having progressive symptoms and signs of heart failure. A preoperative evaluation emphasized the need for cardiac assessment in a cardiology unit. At the time of his admission, the patient complained of fatigue, insomnia, moderate chest tightness, shortness of breath, vomiting, coughing, pain in the lower left limb, and gastrointestinal symptoms (constipation alternating with diarrhoea and bloating).

The patient’s medical history was notable for showing hypertension and an aortic valve replacement (Medtronic Hall tilting disc 23 valve) 22 years prior for severe aortic valve disease due to infective endocarditis. A few years later, vascular surgery for a fissured thrombosed mesenteric artery aneurysm was successfully performed. Moreover, a right ischemic sylvian stroke was noted at the age of 55, a left ischemic sylvian stroke was noted at the age of 59, and intestinal subocclusive syndrome secondary to adhesive small bowel obstruction (ASBO) was diagnosed at the age of 65.

A physical exam revealed a body mass index of 22.1 kg/m^2^; facial hyperaemia; ecchymosis in the upper third of the right forearm; moderate bilateral calf oedema; abolished right basal vesicular murmur; and a painless, uncomplicated right inguinal hernia. Cardiovascular examination identified a regular heart rate of 108 beats per minute, the presence of the opening and closing sounds of the aortic prosthesis, a tricuspid regurgitation murmur, and blood pressure of 115/75 mmHg. Signs of congestion (jugular vein distension and hepatomegaly) were also present.

The laboratory findings were as follows: normocytic normochromic anaemia (haemoglobin of 10.9 g/ dL and a haematocrit value of 31.2%), a white blood count (WBC) of 10,400/μL (85% neutrophils), an international normalized ratio (INR) of 6.1, C-reactive protein of 45.5 μg/mL, an erythrocyte sedimentation rate (ESR) of 69 mm/h, and a N-terminal-prohormone B-Type Natriuretic Peptide (NT-proBNP) value of 6,100 pg/mL. Other tests (blood and nasopharyngeal cultures, routine urine examination) were negative.

The rest electrocardiogram recording displayed a sinus rhythm, left axial deviation, negative T waves in the V5-V6 leads, and low voltage in the limb leads.

Plain chest X-ray revealed right basal pulmonary consolidation associated with mild pleural effusion, an enlarged projection area of the upper and middle mediastinum, cardiomegaly, and aortic atherosclerosis.

**Fig. 1:**
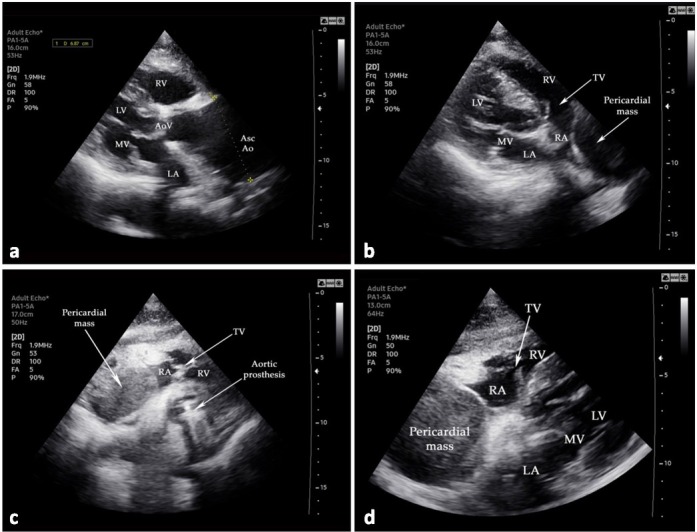
Transthoracic echocardiography examination: (a) the clear presence of the ascending aortic aneurysm (6.87 cm); (b–d) massive pericardial tumoral mass compressing the right atrium. Abbreviations: LA—left atrium; LV—left ventricle; MV—mitral valve; RA—right atrium; RV—right ventricle; SVC—superior vena cava; TV—tricuspid valve; AoV — aortic valve; AscAo — ascending aorta.

Transthoracic echocardiography (TTE) (Samsung HS60 ultrasound machine, Samsung Medison Co., Ltd., Seoul, Korea) showed a 6.87 cm ascending aortic aneurysm, normal left ventricular size and function, and normal functionality of the Medtronic Hall 23 (Medtronic Inc, Minneapolis, Minn, USA) tilting valve. TTE also identified a large hypodense pericardial mass, with a smooth rounded margin and no internal flow detectable, which exerted significant compression of the right atrium but without signs of tamponade (see Figure 1). A strong suspicion of chronic ascending aortic dissection with pericardial hematoma was raised, although the entry tear of the dissection was not detected via TTE.

Ultrasound examination of the abdomen (Samsung HS60 ultrasound machine, Samsung Medison Co., Ltd., Seoul, Korea) was performed, and highlighted the presence of a well-defined 53×65 mm pericardial mass, with minimal ascites, hyperechogenic liver lesions, polycystic kidney disease, and prostatic hyperplasia.

**Fig. 2:**
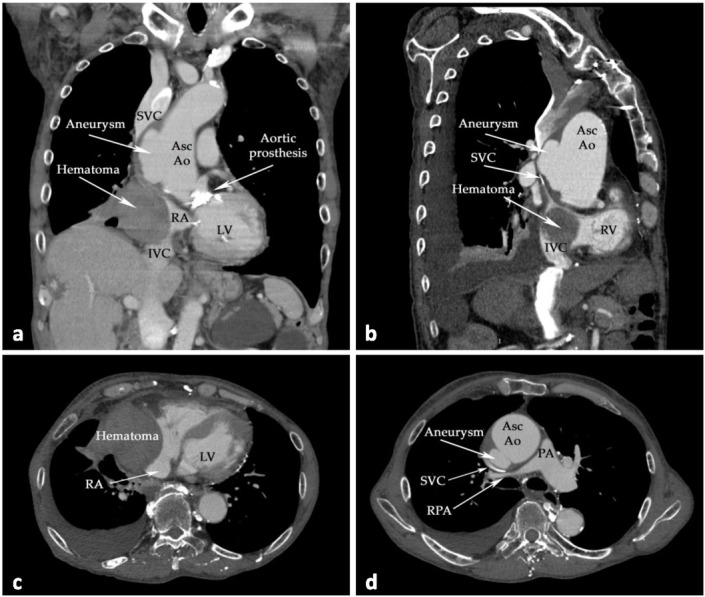
Emblematic thoraco-abdominal CT scans. In the coronal and sagittal scans ((a) and (b), respectively), the presence of an ascending aortic aneurysm, hematoma, and compression on RA, SVC, and IVC can be observed. (c) Compression of RA; (d) compression of SVC and RPA (axial CCTA scans). Abbreviations: AscAo—ascending aorta; IVC—inferior vena cava; LV—left ventricle; PA—main pulmonary artery; RA—right atrium; RPA—right pulmonary artery; SVC—superior vena cava.

Cardiac computed tomography angiography (CCTA) and contrast-enhanced abdominal computed tomography (Iomeron^®^ 350/100 mL, Bracco Imaging S.p.A, Milan, Italy) were subsequently performed (Somatom^®^ Definition AS64, Siemens AG, Munich, Germany). A partially thrombosed ascending aortic aneurysm (63 mm in the axial plane) with ulcerated aortic plaques was identified; the aortic arch and descending aorta (29 mm) were not affected. CCTA also unveiled a solid intrapericardial, well-defined (80×65×70 mm) and delineated, and non-iodophilic tumour with a hyperdense architecture. The tumour exerted marked compression on the right atrium, the superior and inferior caval veins, and the right pulmonary artery (see Figure 2). No paravalvular leak, valve dehiscence, or structural deterioration was observed.

Furthermore, abdominal CT scans showed subcapsular liver haemangioma (segment VIII) and bilateral polycystic kidney disease (maximum diameter 71×56 mm in the left kidney and 34×33 mm in the right kidney).

A pharmacological regimen was then started using carvedilol (Coryol^®^, KRKA, d.d. Novo mesto, Slovenia) spironolactone (Spironolactona Bioeel^®^, Bioeel S.R.L., Targu Mures, Romania), atorvastatin (Atoris^®^, KRKA, d.d. Novo mesto, Slovenia), pantoprazole (Pantoprazol^®^ SUN, Sun Pharmaceutical Industries Europe B.V., Hoofddorp, The Netherlands), intravenous furosemide (Furosemid Zentiva^®^, S.C. Zentiva S.A., Bucharest, Romania) and antibiotics [cefuroxime (Cefuroxima Atb^®^, Antibiotice S.A., Iasi, Romania), and gentamicin (Gentamicina EIPICO^®^, E.I.P.I.Co MED S.R.L., Bucharest, Romania)], and fresh frozen plasma (FFP). The patient did not experience any significant improvement of his symptoms, and the clinical signs of right heart failure persisted.

Preoperative coronary angiogram did not show significant atherosclerotic lesions at the level of the whole coronary tree.

**Fig. 3:**
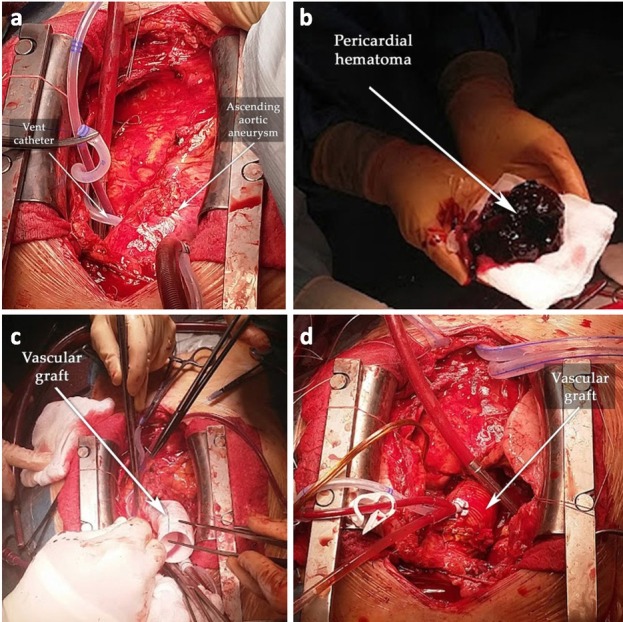
Intraoperative images. (a) Post-sternotomy, the ascending aortic aneurysm was revealed; (b) the chronic pericardial hematoma; (c) the ascending aorta replaced with a 30 mm-diameter vascular graft; (d) the final result.

The patient was then transferred to cardiac surgery. The surgical approach was performed via remedian sternotomy. A total cardiopulmonary bypass (total time: 267 min; aortic cross-clamp time: 135 min) was undertaken with the cannulation of the right femoral artery and vein. A vent catheter was placed through the pulmonary artery (see Figure 3a). The core body temperature was 28°C. For myocardial protection, Bretschneider’s Histidine Tryptophane Ketoglutarate (Custodiol^®^HTK, Essential Pharmaceuticals, Durham, NC, USA) cardioplegic solution was used.

A massive intrapericardial organized hematoma compressing the right atrium, right pulmonary artery, and superior and inferior caval veins was peeled off from the pericardium and surface of the heart (see Figure 3b). A longitudinal supracoronary aortotomy was performed on the anterior aneurysmal wall. A circular dissection flap was observed on the circumference of the aortic wall originating distal to the sinotubular junction. The dissection flap extended on the ascending aorta towards the inferior wall of the aortic arch; the origin of the large vessels was unaffected. The ascending aorta was resected above the sinotubular junction up to 2 cm proximal to the origin of the brachiocephalic trunk and was replaced with a 30 mm-diameter FlowWeave Bioseal^®^ vascular graft (Jotec GmbH, Hechingen, Germany) (see Figure 3c and 3d). The Medtronic Hall 23 aortic valve was in good condition, and no pannus was observed.

Postoperatively, the patient arrived to the ICU intubated and mechanically ventilated and standard monitoring was started. The invasive mechanical ventilation P-SIMV (pressure-synchronized intermittent mandatory ventilation) was continued until the morning of day 1 without any acute events at the moment of extubation. Vasoactive-inotropic support [Dobutamine (Dobutamina^®^, Panpharma Luitre, France) 5 μg/kg/ min + Noradrenaline (Noradrenaline Norepinephrine (tartrate) Aguettant^®^, Laboratoire Aguettant, Lyon, France) 0.2 μg/kg/min, inotropic score of 25], and vasodilators [Nitroglycerine (Nitronal^®^, G. Pohl-Boskamp GmbH&Co. KG, Hohenlockstedt, Germany) 7.5 μg/kg/min], were continued from operating room. Antibiotic therapy [Cefazoline (Zolinef^®^, Medochemie Ltd. (Factory C), Limassol, Cyprus) 6 g/day + Vancomycin (Vancomicina Rompharm^®^, S.C. Rompharm Company S.R.L., Otopeni, IL, Romania) 2 g/day] was started in order to prevent infectious complications.

In the first three days postoperatively the patient experienced an expected normal clinical, hemodynamic and paraclinical evolution, with mean values pCO_2_ of 27.61±5.41 mmHg, pO_2_ of 138.67±52.87 mmHg, pH 7.39±0.08, haemoglobin 7.85±0.85 g/dL, and electrolytes (Na^+^ of 139.36±2.7 mEq/L, K^+^ of 3.89±0.51 mEq/ L). On continuous oxygen mask with a flow of 4-6 l/min, oxygen saturations (SpO_2_) range from 97-99% (98.36±0.79%).

In the third postoperative day, an unexpected cardiopulmonary arrest suddenly occurred. Successful CPR manoeuvres were performed, and P-SIMV was reinitiated for the next 2 hours, continued with pressure support ventilation (PSV) for 8 hours. Later on he was once again extubated without any acute events. The next 2 days in the intensive care unit were uneventful, blood gas values were in physiologic parameters (pCO_2_ 31.06±3.35 mmHg, pO_2_ 145.39±33.45 mmHg, SpO_2_ 98.61±0.61%, pH values of 7.45±0.05). Vasoactive-inotropic support was gradually stopped in day 4 (Norepinephrine) and day 5 (Dobutamine), respectively.

Our patient was discharged after 25 days of hospitalization on a pharmacological regimen consisting of a vitamin K antagonist (Trombostop^®^, Terapia a Sun Pharma Company, Cluj-Napoca, Romania) beta-blockers (Coryol^®^, KRKA, d.d. Novo mesto, Slovenia), a fixed-dose combination (FDC) of sartan and a calcium channel blocker (Twynsta^®^, Boehringer Ingelheim International GmbH, Ingelheim am Rhein, Germany), combined diuretics [spironolactone + furosemide (Diurex^®^, Terapia a Sun Pharma Company, Cluj-Napoca, Romania), and statins (Sortis^®^, Pfizer Manufacturing Deutschland GmbH, Freiburg, Germany).

## Discussions

In older patients, the appearance and progression of HF decades after aortic valve replacement is not uncommon [19,20]. In this population, gradual structural valve deterioration, left ventricular dysfunction, systemic hypertension, coronary artery disease, and arrhythmias are usually the most frequent causes of heart failure.

Routine follow-up is essential for patients after open heart surgery. In our case, the patient discontinued ongoing cardiological checks at least 5 years before admission. No clinical assessments, blood tests, imaging studies, or drug changes were completed in this period. Transthoracic and transoesophageal echocardiographic methods play a key role in the diagnosis of heart failure substrate, assessing cardiac and pericardial structures and their function, and the routine surveillance of prosthetic heart valves, including early and late postoperative complications [19, 20, 21].

Irrespective of the time and modalities of onset, the postoperative complications of AVR can lead to higher rates of morbidity and mortality [22].

Ascending aortic aneurysm can develop later after AVR [23]. Patients with chronic TAAD are frequently asymptomatic, and the precise moment of dissection onset is difficult to define [1]. Previous case reports demonstrated the existence of progressive chronic pericardial hematomas with signs of compression late after the surgical revascularization of the myocardium or aortic valve replacement, respectively [24, 25, 26, 27]. Hirai et al. presented the case of a 63-year-old woman with signs of left HF and chest discomfort associated with CT scans suggestive of the presence of a mass that exerted compression of the heart and left-lower-lung lobe. These findings were found to be associated with a chronic expanding hematoma 14 years after initial heart surgery for a double-outlet right ventricle and eight years after surgery for tricuspid regurgitation [28]. Idiopathic chronic expanding pericardial hematoma was also described as the source of pericardial tamponade in an older male patient [29]. Abugroun et al. presented a case of chronic TAAD with pericardial effusion diagnosed incidentally in a routine preoperative CT abdominal evaluation for elective cholecystectomy in a 63-year-old male [30]. In this study, we present a postoperative TAAD 22 years after aortic valve replacement.

The differential diagnosis of a pericardial mass is challenging, and both benign and malignant (primary or metastatic) diseases should be considered [31]. Heterogenous non-neoplastic pericardial entities (hematomas, thrombi, pseudoaneurysms, cysts, and inflammatory pseudo tumours) are described in the literature [32], as is the contribution of multimodality imaging in the diagnosis of multiple pericardial hematomas and the development of progressive superior vena cava syndrome [33,34]. However, the literature to date has not described the presence of a pericardial haematoma secondary to chronic TAAD compressing the right atrium and the superior and inferior caval veins, leading to right heart failure. Careful TTE or TOE should be performed to differentiate a pericardial mass from an intracardiac thrombus, especially when the limit between the mass and the right atrium wall is not well defined. Echocardiography can also enable the assessment of other cardiac or pleural complications (e.g., aortic regurgitation, prosthetic valve dysfunction, abnormal ventricular wall motion, and pleural effusion). In our case, a high suspicion of pericardial haematoma was raised by TTE used as a routine diagnostic tool for heart failure; no signs of Medtronic–Hall prosthesis-related dysfunction were detected. The presence of a mild right pleural effusion diagnosed by chest X-ray was confirmed via TTE.

The first-line imaging modality in the setting of aortic diseases (acute or chronic) is computed tomography [35]. The excellent diagnostic performance of CCTA (98–100% specificity/sensibility) is well-established in the evaluation of complete aortic anatomy, including the aorta’s branches and periaortic structures [36]. The ECG-gated CCTA findings in this case (i.e., the presence of chronic haematoma with signs of right atrial and bicaval compression and limited antegrade dissection with respect to the origin of the brahiocephalic trunk) confirmed and fulfilled the initial TTE data and helped the cardiac surgeon to determine the best approach. Indeed, the correct aortic cannulation and clamp site and replacement of the ascending aorta above the sinotubular junction with the FlowWeave Bioseal**^®^** vascular graft were orientated via CCTA.

Chronic TAAD cases are surgically treated in approximatively 60% of cases during follow-up [37]. In our case, the confirmed diagnosis of a concomitant pericardial haematoma with signs of compression on surrounding structures required an emergency surgical procedure to be performed.

There are, however, some concerns regarding the long-term outcome of this case related to the multifactorial patient characteristics (gender, age, dyslipidaemia, complex cardiovascular reintervention, concomitant HF, and hypertension).

The management of patients with TAAD and mechanical aortic valves is demanding. An antithrombotic regimen is a continuous challenge; the rate of thromboembolic events in all patients with mechanical heart valves has been reported to be 2.5 to 3.7% [38]. Presently, in patients with mechanical heart valves, all DOACs (direct thrombin inhibitors or factor-Xa inhibitors) are contraindicated, even though positive results were reported in some small exploratory studies, and laboratory methods for determining the plasmatic concentrations of apixaban and rivaroxaban have been developed [38, 39, 40, 41, 42]. An effective, rigorous, long-life anticoagulation regimen with a vitamin K antagonist is essential [43,44]. Acenocumarol was prescribed in adjusted doses to obtain and maintain efficient anticoagulation (INR: 2.5–3.0).

The treatment of coexisting heart failure and hypertension requires an individualized regimen. Beta blockers and ACE inhibitors/sartans are strongly indicated in the treatment of heart failure and have beneficial effects on mortality in acute TAAD [19,45]. Based on these recommendations, we decided to use adjusted doses of carvedilol. A combined regimen with a mineralocorticoid receptor antagonist (spironolactone), a diuretic (furosemide), and a FDC of telmisartan+amlodipine was also recommended, in line with current literature [19,46].

Statins have well-recognized positive effects in atherosclerotic cardiovascular diseases. Their influence on mortality in acute and chronic TAAD or thoracic aortic aneurysms, however, has rarely been investigated. Nevertheless, some studies suggest a better prognosis [47, 48, 49, 50]. For our patient, a high dose of atorvastatin (80 mg) was added to the above-mentioned drugs.

## Conclusions

The clinical diagnosis of right heart failure related to a pericardial hematoma compressing the right atrium and caval veins is highly improbable. Multimodal imaging techniques (echocardiography and ECG-gated CCTA) are essential for accurate diagnosis and patient-oriented treatment, particularly in cases with previous heart valve surgery and atypical stable haemodynamic presentation. The long-term follow-up of such cases reinforces the need for a permanent adjusted medical regimen combined with staged imagistic studies. Further research addressed to the individual positive effects of cardiovascular drugs, including oligodeoxynucleotidesis-based therapies are needed to establish effective approaches for treating the development and progression of aortic aneurysms.
